# Severe Hypercholesterolemia: A Unique Presentation of Non-Hodgkin's Lymphoma in a Patient with Neurofibromatosis Type 1

**DOI:** 10.1155/2014/579352

**Published:** 2014-07-01

**Authors:** Kenechukwu Chudy-Onwugaje, Nnaemeka Anyadike, Yuriy Tsirlin, Ira Mayer, Rabin Rahmani

**Affiliations:** ^1^Department of Internal Medicine, Maimonides Medical Center, Brooklyn, NY 11219, USA; ^2^Department of Gastroenterology, Maimonides Medical Center, Brooklyn, NY 11219, USA

## Abstract

We report a case of non-Hodgkin's lymphoma (NHL) with an unusual initial manifestation as severe hypercholesterolemia and obstructive jaundice in a patient with neurofibromatosis type 1 (NF 1). NHL should be considered in the evaluation of obstructive jaundice alone or in combination with severe hypercholesterolemia. Relief of biliary obstruction led to the resolution of hypercholesterolemia in our 59-year-old male patient, followed by doxorubicin-based chemotherapy for the underlying lymphoma. NF 1 is a genetic condition that results from a defect in a tumor-suppressor gene and it is likely that this led to the development of NHL in our patient. It is important that clinicians are familiar with the gastrointestinal manifestations of NF 1, especially its association with intra-abdominal malignancies, when treating patients with a personal or family history. To the best of our knowledge, this is the first case of NHL presenting initially as severe hypercholesterolemia and it is also one of the few instances where NHL has been reported in association with NF 1.

## 1. Introduction

Elevation in serum total cholesterol level to values greater than 1,000 mg/dL is extremely rare and has only been described in a limited number of medical conditions. Obstruction of the biliary tract is a rarely encountered cause of this degree of hypercholesterolemia and one of the first descriptions of this association was made by McIntyre et al. in 1975 [1]. They reported markedly elevated serum cholesterol levels in two patients presenting with jaundice, noting a difference in their lipoprotein profile compared to that seen in hypercholesterolemia from other causes [1].

Neurofibromatosis type 1 (NF 1, von Recklinghausen disease) is an uncommon autosomal dominant genetic disorder occurring at a prevalence of 1 in 3,000 live births and causing a spectrum of clinical manifestations, including a predisposition to tumor formation [2]. However, it has scarcely been known to be associated with the development of malignant lymphomas. We report a case of non-Hodgkin's lymphoma (NHL) in the porta hepatis presenting initially with severe hypercholesterolemia and obstructive jaundice, causing a diagnostic dilemma in a patient with NF 1. This is the first report in the English literature of the unusual presentation of non-Hodgkin's lymphoma with severe hypercholesterolemia and is one of a few instances of NHL manifesting initially with obstructive jaundice or in association with NF 1 [3, 4]. Non-Hodgkin's lymphoma should be considered in the differential diagnosis of severe hypercholesterolemia and obstructive jaundice, especially in patients with tumorigenic conditions like NF 1.

## 2. Case

A 59-year-old Hispanic male presented to the emergency room with a 1-month history of episodic epigastric pain, pruritus, a 15-pound weight loss, and worsening jaundice. On physical examination, he was deeply icteric and vital signs were as follows: pulse rate, 118/min; respiratory rate, 20/min; blood pressure, 116/80 mm/Hg; and body temperature 98.3 F. Abdominal examination revealed mild distension, an enlarged nontender liver, and extensive skin excoriations. He also had striking cutaneous features meeting the criteria for NF 1 with multiple skin fibromas greater than 10 in number, café-au-lait spots greater than 1.5 cm in diameter, and extensive axillary freckling (D). He was 32 years old at the appearance of the first skin lesion and his condition appears to have been sporadic as he had no preceding family history of neurofibromatosis.

**Figure 1:**
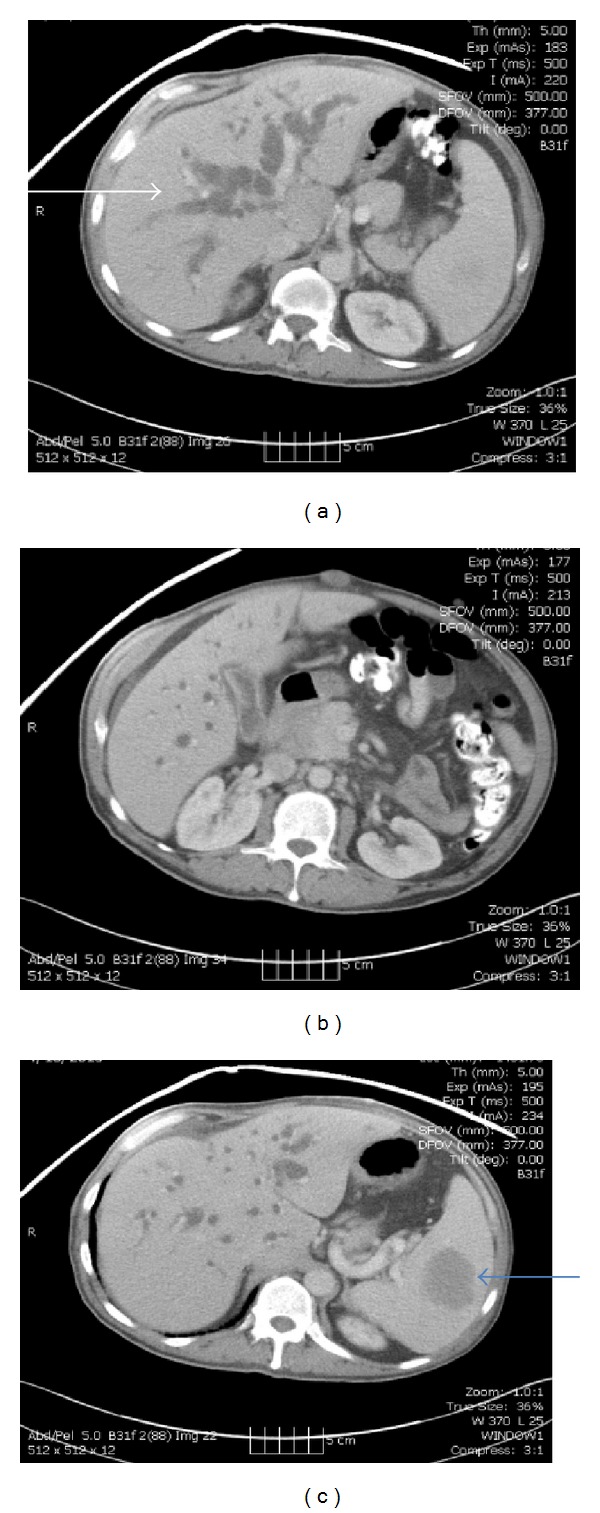
Computed tomography scans of the abdomen showed the porta hepatis mass, markedly dilated intrahepatic bile duct (white arrow), a 5 cm splenic mass (blue arrow), and a retroperitoneal mass.

**Figure 2:**
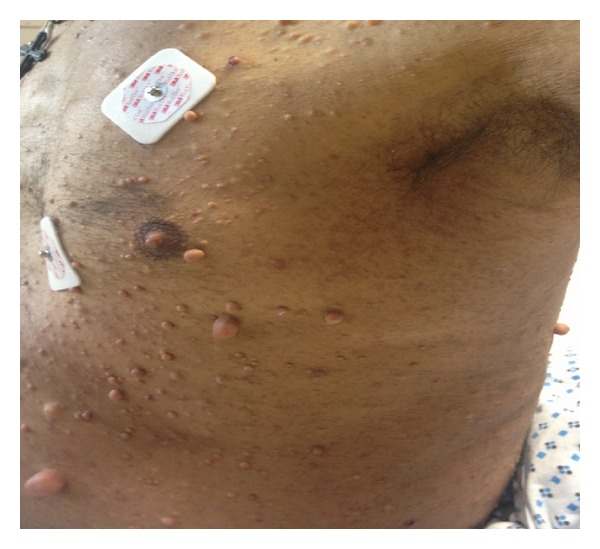
Subcutaneous neurofibromas and axillary freckling as noted in the patient at the time of admission.

Initial laboratory studies revealed normal blood counts and a cholestatic liver profile as shown in Table 1. Abdominal ultrasonography showed marked intrahepatic and extrahepatic biliary dilatation, with the proximal portion of the common bile duct (CBD) measuring 17 mm in diameter, and computerized tomography (CT) revealed a 4.5 cm × 5 cm × 3.4 cm hypoenhancing soft tissue mass in the porta hepatis surrounding the portal vein and the common hepatic artery and extending to the head of the pancreas (Figures 1(a) and 1(b)). The pancreatic duct was of normal caliber on magnetic resonance cholangiopancreatography (MRCP) but an abrupt occlusion was seen in the middle portion of the common bile duct. Besides the porta hepatis tumor, additional masses were also observed on imaging: a 5 cm hypoenhancing mass in the spleen (Figure 1(c)), a 4.5 cm × 4.5 cm × 2.8 cm hypodense mass in the retroperitoneum, and multiple raised skin lesions consistent with the subcutaneous fibromas of NF 1 (Figure 2). CA 19-9 was elevated at 200 u/mL (reference range, less than 34 u/mL) but carcinoembryonic antigen (CEA) was within normal range.

As preparations were underway for endoscopic biliary decompression on the second day of admission, a routine lipid panel was obtained and the results were startling: total cholesterol, greater than 1,000 mg/dL; HDL cholesterol, 19 mg/dL; triglycerides, 233 mg/dL; and LDL cholesterol, greater than 1,000 mg/dL. A repeat test to rule out clinical or laboratory errors confirmed this abnormal result. During endoscopic retrograde cholangiopancreatography (ERCP), a tight 6 cm stricture was noted in the proximal common bile duct (CBD) on cholangiogram with greatly dilated intra- and extrahepatic ducts. Biliary sphincterotomy was performed with successful deployment of self-expanding metallic stents across the stricture for biliary decompression. Linear endoscopic ultrasound (EUS) showed the porta hepatis mass to be encasing the CBD and abutting the portal vein. Following the relief of biliary obstruction, jaundice improved and cholesterol levels declined steadily. He was discharged home and at a follow-up visit three months later, total cholesterol, triglyceride, and LDL and HDL cholesterol levels were close to normal at 208 mg/dL, 110 mg/dL, 128 mg/dL, and 55 mg/dL, respectively. Pathology from the splenic mass revealed a low-grade follicular lymphoma while that from the retroperitoneal mass revealed a peripheral nerve sheath tumor consistent with a neurofibroma and cells that were S100(+), CD34(+), SMA(−), and CD117(−).

Because of the precarious location of the porta hepatis mass, intimately associated with the porta hepatic vessels and CBD, there were initial safety concerns regarding biopsy of this mass. However, the patient had a massive gastrointestinal bleed three months later that did not respond to endoscopic or radiologic interventions and needed open surgery. A gastrostomy with oversewing of the gastroduodenal artery was performed to control the bleeding and surgical biopsy of the porta hepatis mass and liver was obtained. Pathology of the porta hepatis mass revealed a diffuse large B-cell lymphoma positive for the CD 10, CD 19, CD 20, and CD 22 markers, and examination of liver tissue showed bile duct proliferation consistent with biliary obstruction and marked fibrosis. On positron emission tomography (PET) scan, there was intense uptake in the spleen, porta hepatis, and the distal stomach and surrounding the proximal duodenum without abdominal or pelvic adenopathy. He has since been started on rituximab and a reduced-dose CHOP (rituximab, cyclophosphamide, doxorubicin, vincristine, and prednisone) chemotherapy regimen and is responding well to treatment.

## 3. Discussion

The occurrence of severe hypercholesterolemia in clinical practice is rare and its workup can be confusing to the clinician. It is often encountered as a component of genetic disorders like familial hypercholesterolemia but less commonly as a result of biliary obstruction [5]. Obstructive causes reported in the literature to produce hypercholesterolemia include pancreatic carcinoma and primary biliary cirrhosis, but it has not been known to be associated with non-Hodgkin's lymphoma [6, 7]. NHLs are a heterogeneous group of lymphoproliferative disorders typically affecting nodal and extranodal sites and producing a myriad of nonspecific clinical features. When it affects the liver, obstructive jaundice is a rare and late manifestation, often resulting in death within three months of onset [3]. The etiology of jaundice in NHL is most commonly due to tumor compression of the extrahepatic bile duct as in our patient, but intrahepatic cholestasis and parenchymal involvement are possible [3, 8]. Severe hypercholesterolemia in association with obstructive jaundice as seen in this case has never been reported in NHL, much less as a presenting feature of this malignancy. Because the index patient had no personal or family history of hypercholesterolemia, its discovery on the second day of admission posed a diagnostic dilemma as it was not clear if the dyslipidemia was a separate entity or related to the unknown cause of his obstructive jaundice. Additionally, the rare association of obstructive jaundice with NHL further short-sighted our list of differentials.

The hypercholesterolemia seen in cholestasis is unique as the lipoproteins typically have an aberrant composition, with lipoprotein-X (LpX) having been identified as the primary abnormal “obstructive” lipoprotein [1]. LpX has an unusual electrophoretic mobility and is rich in phospholipids and unesterified cholesterol, with very little triglyceride and protein makeup. Lipoprotein-Y is another lipoprotein rich in triglyceride and fractionating in the LDL range which has also been identified in biliary obstruction [9]. These abnormal lipoproteins in cholestasis contribute to increased cardiovascular risk and their presence in serum can produce false laboratory results leading to delays in medical management [6, 7]. The precise mechanism by which cholestasis leads to hypercholesterolemia is not clear but several theories abound, including one informed by the findings of Fredrickson et al. in rats where CBD ligation led to an increase in hepatic cholesterol synthesis [10]. Biliary decompression is a mainstay of treatment of cholestatic hypercholesterolemia; our patient's serum cholesterol levels returned to normal following stent placement [9]. Nonetheless, the underlying obstructive cause should be addressed and, for NHLs, use of doxorubicin-containing chemotherapy regimen is regarded as ideal. We used a reduced dose of doxorubicin for our patient because it is excreted in bile as we had noted a mild elevation in his liver enzymes before the initiation of chemotherapy. However, delaying treatment with doxorubicin for one or two cycles may be prudent in patients with obstructive jaundice who are at increased risk for liver toxicity [11].

In our case, it is likely that the patient's personal history of neurofibromatosis predisposed him to the development of NHL. Non-Hodgkin's lymphoma occurring in patients with NF 1 has been scarcely reported and adds to the rarity of the observations made in this one patient [4, 12]. Neurofibromatosis type 1 occurs as a result of a defect in the NF 1 tumor suppressor gene on chromosome 17q11.2 and its diagnosis is made when two or more predefined clinical measures are met [13, 14]. NF 1 encodes a cytoplasmic protein known as neurofibromin that controls cellular proliferation and is inherited in an autosomal dominant fashion with variable expressivity [13]. The clinicopathologic consequences of NF 1 are numerous and its intra-abdominal manifestations can be seen at any site in the gastrointestinal tract and in the retroperitoneum and can rarely include lymphomas as seen in our patient [13, 15]. These intra-abdominal manifestations usually arise later in life after the cutaneous manifestations of the disease, like in our case where the skin features predate the gastrointestinal symptoms by more than 20 years [13].

This case is unique because of the unusual initial presentation of NHL and its association with neurofibromatosis—a combination of rarities. Clinicians should strongly consider NHL in the differential workup of patients presenting with obstructive jaundice alone or in combination with severe hypercholesterolemia. Relief of obstruction is necessary for resolution of severe hypercholesterolemia and doxorubicin-based chemotherapy is the standard for the treatment of NHL. Notably, the gastrointestinal manifestations of NF 1 are underrecognized and clinicians treating patients with a personal or family history of NF 1 should be aware of its numerous presentations—especially its association with intra-abdominal malignancies.

## Figures and Tables

**Table 1:** Pertinent laboratory values on admission.

Laboratory parameter	Value on admission	Reference range
Total bilirubin	18.6 mg/dL	0.4–1.1 mg/dL
Direct bilirubin	12.8 mg/dL	0.1-0.2 mg/dL
Aspartate aminotransferase	133 U/L	14–34 U/L
Alanine aminotransferase	68 U/L	7–58 U/L
Alkaline phosphatase	Greater than 1650 U/L	31–105 U/L
White blood cell count	8,000/mL	4,000–10,800/mL
Hemoglobin	14.5 g/dL	14.0–18.0 g/dL
Platelet count	256,000/mL	150,000–500,000/mL
International normalized ratio	1.4	0.9–1.2
